# Effects of Chronic Musculoskeletal Pain on Fertility Potential in Lean and Overweight Male Patients

**DOI:** 10.1155/2017/4628627

**Published:** 2017-12-24

**Authors:** Fereshteh Dardmeh, Hiva Alipour, Hans Ingolf Nielsen, Sten Rasmussen, Parisa Gazerani

**Affiliations:** ^1^Department of Health Science and Technology, Biomedicine Group, The Faculty of Medicine, Aalborg University, Aalborg, Denmark; ^2^SMI®, Department of Health Science and Technology, The Faculty of Medicine, Aalborg University, Aalborg, Denmark; ^3^Department of Clinical Medicine, The Faculty of Medicine, Aalborg University Hospital, Aalborg, Denmark

## Abstract

Both chronic pain and obesity are known to affect reproductive hormone profiles in male patients. However, the effect of these conditions, alone or in combination, on male fertility potential has received less attention. 20 chronic musculoskeletal pain patients and 20 healthy controls were divided into lean and overweight subgroups according to their BMI. Current level of chronic pain (visual analogue scale) and pressure pain thresholds (PPTs) in 16 predefined sites, classically described and tested as painful points on the lower body, were measured. Levels of reproductive hormone and lipid profiles were assessed by ELISA. Sperm concentration and motility parameters were analyzed using a computer-aided sperm analysis system. Sperm concentration, progressive motility, and percentage of hyperactivated sperm were generally lower in the chronic pain patients in both lean and overweight groups. The overweight control and the lean chronic pain groups demonstrated a significantly lower percentage of progressively motile sperm compared with the lean control group, suggesting that musculoskeletal chronic pain may have a negative influence on sperm quality in lean patients. However, due to the potential great negative influence of obesity on the sperm parameters, it is difficult to propose if musculoskeletal chronic pain also influenced sperm quality in overweight patients. Further research in chronic pain patients is required to test this hypothesis.

## 1. Introduction

Male factor infertility accounts for approximately 40% of infertility cases [1, 2]. Assessment of semen quality is the first step in assessing male factor infertility [3, 4] and depends on several parameters including semen volume, sperm concentration, total count of spermatozoa, motility, vitality, and morphology [5]. Increased BMI has been suggested to negatively affect spermatogenesis and the molecular structure of germ cells in the testis and sperm maturation in the epididymis [6, 7], consequently resulting in reduced sperm quality and male subfertility [8–12].

The World Health Organization (WHO) defines overweight and obesity as excessive or abnormal fat accumulation, which impairs health [13]. The body mass index (BMI) is the most frequently used assessment for overweightness and obesity [14]. The WHO has defined a BMI greater than 25 kg/m^2^ as overweight and a BMI greater than 30 kg/m^2^ as obese [15].

In contrast to the extensive attention paid to the effect of obesity on many health-related issues, the impact of obesity on reproduction has received less attention [8, 9]. Obesity has been reported to increase the conversion of androgens to estradiol, leading to elevated levels of serum estrogen; the increased estradiol in turn impacts the hypothalamus, causing stimulation of GnRH, to release FSH and LH, resulting in reduced testicular function and reduced production of testosterone (both intratesticular and circulating testosterone) [8]. Infertile men have demonstrated a lower testosterone and higher estradiol levels compared with fertile men, which may be due to the gonadotropin suppression by estradiol and can be considered as a common marker for fertility potential in men [16]. It is also suggested that excessive estradiol has a direct negative effect on spermatogenesis [8, 16].

The role of reproductive hormones in pain is not well understood. Many hormonal effects on pain which were initially thought to be due to gonadal release of the hormone may actually be due to the hormone synthesis in tissues such as the brain and spinal cord (e.g., progesterone and estradiol). Many of the central regions implicated in pain and analgesia contain receptors for both estrogens and androgens and have the ability to synthesize steroids locally. Binding of specific steroids to their receptors within these central and peripheral regions is likely involved in ligand-dependent transcriptional events, thereby influencing the expression of various neurotransmitters and receptors. It has been suggested that sex steroids have profound effects not only on developmental organization but also on the ongoing dynamics of the nervous system [17, 18].

Musculoskeletal disorders (MSDs) are generally associated with pain due to a sensory disturbance, caused by proinflammatory conditions [7]. The development and progression of the MSDs are thought to be through increased mechanical load and altered metabolic and hormonal changes driven by overweightness and obesity [13, 19]. A full understanding of the pathological mechanisms underlying the effects of obesity on musculoskeletal disorders requires further investigation [7, 19].

Neuroplasticity is the alteration of neural pathways and synapses due to changes in behavior, environment, and neural process as well as changes resulting from physical injury like obesity [19–21]. Neuroplasticity can also result in the change of nerve cells, pain receptors, and the surroundings of the nerve signal due to the release of inflammatory substances associated with primary tissue damage [20–22]. McVinnie suggested that pain and obesity are more often two sides of the same coin as chronic pain can also lead to obesity because of inactivity or perhaps a genetic factor contributing to both [19].

Obesity can affect chronic pain, and a link between obesity and semen quality exists. Considering that both obesity and chronic pain can affect the reproductive hormonal profiles in male patients, this study aimed to assess and compare reproductive hormones and semen quality in overweight and normal weight chronic pain patients and healthy controls to provide a deeper insight into the possible relation between obesity, chronic pain, and male fertility potential.

## 2. Materials and Methods

This study was performed at the orthopedics department, Aalborg University Hospital, and Fertility Clinic in Dronninglund, Denmark, from June 2014 to December 2015, following approval from the Scientific Ethics Committee of the Northern Jutland Region, Denmark (approval reference no. N-20140025).

### 2.1. Study Population

Patients attending the orthopedics department of Aalborg University Hospital (Aalborg, Denmark), with a history of at least 3 months of low back pain and lower extremity chronic pain with a minimum score of three on the visual analogue scale (VAS), were considered and invited by the study healthcare professional to take part in this study as the test group. Healthy volunteers with no previous history of chronic musculoskeletal pain were also recruited from males visiting the clinic for checkups or as accompanying persons. Age (18–46 years) and BMI (lean < 25 kg/m^2^ < overweight) were initially considered to recruit a sufficient number of patients for all study groups.

All participants provided written consent and filled a questionnaire describing the history of the chronic pain and information regarding possible confounding factors (i.e., medication, reproductive surgery, or complications), which were taken into account for the patients' inclusion and assessment of results. Based on the approved protocol, participants using medications including strong analgesics (e.g., opioids), having an ongoing infection, with current or previous neurologic, musculoskeletal, or mental illnesses, malignancy, drug addiction (cannabis, opioids, or other psycho drugs), reproductive surgery, and related complications were not included in the study.

Height and weight were measured to calculate the body mass index (BMI). A BMI ≥ 25 kg/m^2^ was considered as overweight in this study. Recruitment was continued until each of the chronic pain and control groups had equal numbers (10) of participants in the overweight and lean groups (determined based on BMI).

The subgroups (2 × 2 design) based on BMI and pain status (10 patients in each group) are as follows:

- Overweight patients with chronic pain (OP)
- Overweight patients without chronic pain (OC)
- Lean patients with chronic pain (LP)
- Lean patients without chronic pain (LC).

**Figure 1:**
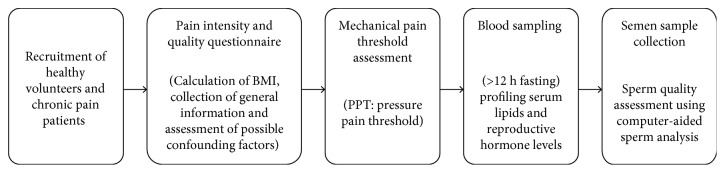
Schematic timeline demonstrating the order procedures performed in the study.

The mean (±SD) and median (min–max) age and BMI of the participants have been demonstrated in Table 1.  Figure 1 illustrates the order of the performed tests and procedures in a schematic timeline.

### 2.1.1. Pain Intensity and Quality Assessment

The chronic pain patients were asked to rate their minimum and maximum pain intensity at rest condition during the previous three months, by marking on a 10 cm horizontal visual analogue scale (VAS) of 0–10 (0 = “no pain”; 10 = “the most pain imaginable”). The updated Short Form McGill Pain Questionnaire (SF-MPQ-2) [23] was used to assess the pain quality. The total score and its 4 subscale scores (continuous pain, intermittent pain, predominantly neuropathic pain, and affective descriptors) of the SF-MPQ-2 questionnaire have been validated as a reliable, valid, and responsive tool for the assessment of pain in patients with chronic pain, acute low back pain, and associated radicular leg pain [24]. A body chart was also provided to each subject in order to mark the distribution area of perceived pain.

### 2.1.2. Mechanical Pain Threshold Assessment

**Figure 2:**
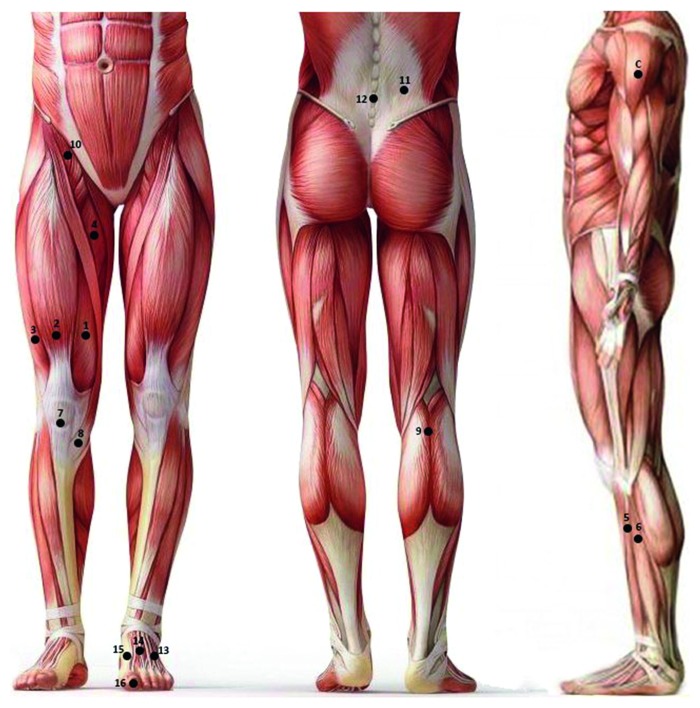
The anatomic sites for pressure pain threshold evaluations over the muscles, patellar tendon, pes anserinus bursae, and supraspinous ligaments in the anterior, posterior, and lateral views. 1: vastus medialis muscle; 2: rectus femoris muscle; 3: vastus lateralis muscle; 4: adductor longus muscle; 5: tibialis anterior muscle; 6: peroneus longus muscle; 7: patellar tendon; 8: pes anserinus bursae; 9: popliteous muscle; 10: iliacus muscle; 11: quadrates lumborum muscle; 12: supraspinous ligaments, area between L5–S1 and S1–S2; 13, 14: extensor digitorum brevis; 15: extensor hallucis longus; 16: flexor halluces longus; C: deltoids.

Quantitative sensory testing paradigms such as pressure pain threshold (PPT) mapping by dynamic pressure algometry have been used to assess the functional status of the excitability of the pain system and to describe mechanical pain sensitivity in larger areas of the body covering one or more muscles [25–27]. Mechanical pain thresholds were assessed in both chronic pain and control groups by a handheld pressure algometer (Somedic, Sweden) in predefined points, which have been classically described and tested as painful areas [28, 29] (Figure 2), and one reference point located on the upper arm of the nondominant hand which was not affected by the pathogenesis of the disease [30] was considered for pressure pain threshold (PPT) measurements.

A gradually increasing pressure was applied to the predefined points by the investigator, while subjects were instructed to press the stop button to record the PPT at “the point where the pressure sensation just becomes painful.” A display on the algometer aided the investigator to deliver a constant increasing pressure (30 kPa/s), with the algometer tip (1 cm^2^ probe area) applied perpendicularly at the stimulation site. The procedure was repeated three times with an interval of 1 min for all test points. Averages of the three repetitions were considered as the subject's PPT for each test point and used for further analysis.

### 2.1.3. Blood Sampling for Profiling Serum Lipids and Reproductive Hormone Levels

All participants were asked to fast for ≥12 hours prior to attending the assessment sessions. Blood samples from all participants were collected from the median cubital, cephalic, or basilic veins by an experienced technician at the laboratory of clinical biochemistry at Aalborg University Hospital (Aalborg, Denmark) and assessed for lipid profiles (triglyceride, total cholesterol, LDL, VLDL, and HDL), luteinizing hormone (LH), and follicle-stimulating hormone (FSH) according to the routine protocols. Testosterone levels were analyzed and reported by the “Statens Serum Institut (SSI)” (Copenhagen, Denmark).

### 2.2. Semen Collection and Analysis

Semen samples were collected by masturbation and allowed to liquefy for 30–45 minutes at room temperature. Following liquefaction, the total volume of each sample was measured using a graduated pipette, before being divided into three parts to be used for the following assessments.

#### 2.2.1. Concentration, Motility, and Kinematic Parameters

Five microliters of the liquefied sperm suspension was loaded into a “Leja chamber slide” (20 *μ*m deep) (Leja Products B.V., Nieuw-Vennep, Netherlands) and placed on a temperature-controlled stage (37°C) of the Nikon E50i microscope, equipped with a 10× positive phase-contrast objective (total magnification of 100x) in conjunction with a phase-contrast condenser and a Basler sca780 (Basler, Germany) camera. Samples were assessed for concentration and motility (kinematic parameters) using the motility/concentration module of the Sperm Class Analyzer (SCA®, Ver. 5.4, Barcelona, Spain) computer-aided sperm analysis (CASA) system.

The SCA motility module provided detailed velocity and motion-path kinematic parameters. The default cutoff values of the SCA were used to assess the following motion parameters as also specified by the WHO (WHO [31]): curvilinear velocity (VCL, *μ*m/s), straight line velocity (VSL, *μ*m/s), average path velocity (VAP, *μ*m/s), amplitude of lateral head displacement (ALH, *μ*m), linearity (LIN), wobble (WOB), straightness (STR), and beat-cross frequency (BCF, Hz) defined at 50 fps. Based on these assessments, the sperm motility was classified as rapid (VCL > 25 *μ*m/s), medium (25 < VCL < 5), slow (VCL < 5), and immotile (VCL = 0 *μ*m/s) and sperm progression as progressive motile (PM, STR > 80%), nonprogressive motile (NPM, 80% > STR > 0%), and immotile (immotile, STR = 0%).

The percentage of hyperactivated spermatozoa was assessed based on the VCL, LIN, and ALH parameters at 50 fps.

#### 2.2.2. Morphology

Air-dried sperm smears were prepared and stained using “Spermblue” (Microptic S/L, Barcelona, Spain) according to the manufacturer's instructions (10 minutes in fixative solution followed by 10 minutes in sperm blue stain) before being assessed using the morphology module of the SCA (automatically quantified measurements of sperm head and midpiece) at 1000x magnification.

#### 2.2.3. DNA Fragmentation (Halosperm Kit)

The DNA fragmentation of the samples was assessed using the Halosperm kit (Halotech, Madrid, Spain) according to the manufacturer's instructions. In general, unfixed sperm cells are immersed in an agarose microgel on a slide incubated in an acid unwinding solution that transforms DNA breaks into single-stranded DNA, followed by immersion in a lysing solution to remove protamines. After staining, the spermatozoa without fragmented DNA show stained nucleoids with big halos of spreading of DNA loops, whereas those with fragmented DNA appear with a small or no halo. The halo sizes and fragmentation index were assessed using the “SCA DNA fragmentation” module.

#### 2.2.4. Statistics

Data are shown as mean ± standard deviation (SD) unless stated differently. All data were checked for normal distribution, and parametric tests were applied. For nonnormal cases, either the Kruskal–Wallis nonparametric test was used or data were corrected using logarithmic transformation prior to further assessments. Multivariate two-way analysis of variance (MANOVA) was used to compare differences in weight, lipid profile parameters (cholesterol triglyceride, HDL, and LDL), hormones (LH, FSH, and testosterone), and semen kinematic parameters in a full factorial model with pain group (pain and no pain) and weight group (lean and overweight) as the factors in this model. A *P* value of ≤0.05 was considered significant. The “IBM^®^ SPSS^®^ Statistics” (Ver. 23, IBM, USA) was used to perform statistical analysis.

## 3. Results

All participants completed the study and no safety issues were reported or recorded following the procedures applied in the study sessions.

### 3.1. Pain Intensity and Quality

Overweight and lean chronic pain patients reported their minimum pain intensity levels of 5.15 ± 2.21 and 4.7 ± 1.97 cm and maximum peak pain intensities of 7.78 ± 1.97 and 8.01 ± 1.77 cm on the VAS, respectively, which corresponded to their pain within the last 3 months.

**Figure 3:**
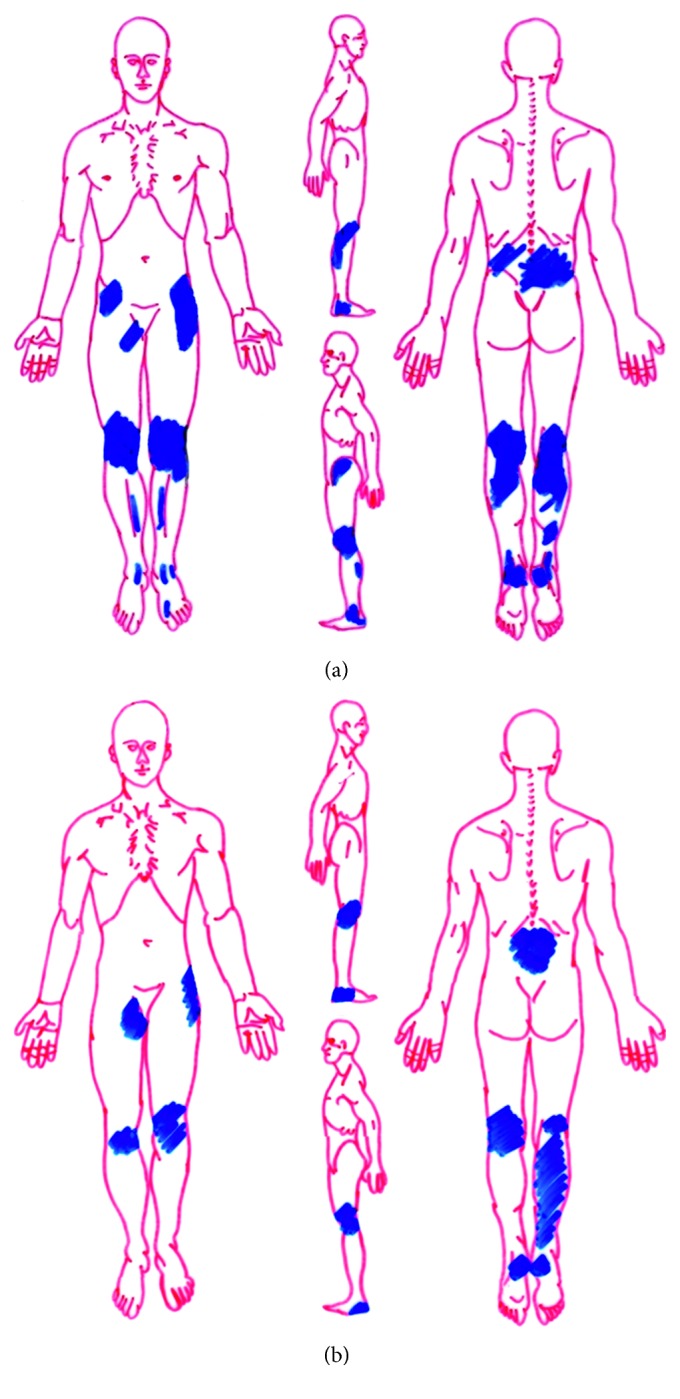
Superimposed pain distribution areas as indicated in OP (overweight chronic pain, (a)) and LP (lean chronic pain, (b)) patients.

Figure 3 demonstrates the distribution of the pain in the chronic pain patients. The following terms were the most reported (30%) descriptors of pain by the chronic pain subjects: throbbing, shooting, boring, sharp, hot, taut, exhausting, and grueling, blinding, and nauseating.

No statistically significant difference in pressure pain thresholds (PPTs) was observed in any of the assessed spots between the subgroups. However, pain groups (LP and OP) demonstrated a trend towards lower pain thresholds compared with the control groups (LC and OC) (Table 2).

### 3.2. Semen Analysis

#### 3.2.1. Concentration, Motility, and Sperm Kinematic Parameters

Semen analysis parameters were assessed using the SCA, and the results are summarized in Table 3.

Both lean and obese control groups demonstrated insignificantly higher mean concentrations compared with their respective chronic pain groups.

The LC group demonstrated a significantly higher percentage of progressively motile sperm (*p=0.034*) and consequently a lower percentage of immotile and nonprogressively motile sperm compared with the LP group. The percentage of sperm in the different motility categories in the OC and OP groups demonstrated a similar trend however insignificant (Table 3).

Moreover, BMI demonstrated a significant influence on the percentage of sperm with progressively motility among both lean and overweight groups (*p=0.036*) (Table 3). The interaction between pain and weight groups was significant for progressive motility (*p=0.02*), suggesting that the effect of pain level on the percentage of progressively motile sperm was different between the different weight groups.

Chronic pain demonstrated a significant influence on VCL, STR, and WOB in overweight patients, with significantly decreased (*p=0.049*, *p=0.025*, and *p=0.021*, resp.) values in OP compared with OC. The LC group demonstrated the same tendency when compared with LP; however, no statistical significance was found (Table 4).

VCL also showed a significantly (*p=0.008*) lower value in the OC compared with the LC, while STR and WOB demonstrated significantly lower values (*p=0.026* and *p=0.006*) in the OP compared with the LP group. The interaction between pain and weight groups was significant for VCL, LIN, and WOB (*p=0.02*, *p=0.05*, and *p=0.01*, resp.), suggesting that pain level affected these parameters differently based on the weight group.

#### 3.2.2. Morphology and DNA Fragmentation

There was no significant difference in the morphology and DNA fragmentation in lean and overweight chronic pain patients compared with the healthy controls. Both lean and obese chronic pain groups displayed a trend towards higher percentage of DNA fragmentation and lower percentage of normal morphology compared with the respective control groups (Table 3), but the interaction level between pain and weight groups was not significant for morphology or DNA fragmentation parameters (*p=0.88* and *p=0.20*, resp.).

### 3.3. Blood Serum Lipid Profile and Reproductive Hormones

**Figure 4:**
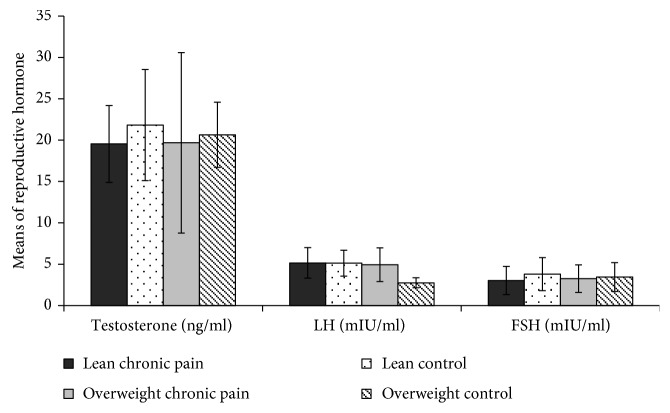
Estimated marginal means of reproductive hormones in overweight pain patient (OP), overweight control patient (OC), lean pain patient (LP), and lean control patient (LC). Provided values are mean ± standard deviation. Multivariate two-way analysis of variance (MANOVA) was used to compare differences in lipid profile. None of the ANOVA results are significant.

The plasma testosterone levels remained within the reference ranges for the hormone (10.3–27.4 nmol/L) (“Testosterone (R-nr. 515)—Statens Serum Institut,” 2015) and did not show any significant difference between the lean and overweight chronic pain patients and their healthy controls. Both chronic pain (LP and OP) subgroups demonstrated a tendency towards lower average values of FSH and higher values of LH compared with the control group (Figure 4). Testosterone, FSH, and LH hormone levels did not show a significant interaction between pain and weight groups (testosterone: *p=0.87*, FSH: *p=0.63*, and LH: *p=0.87*).

There was a tendency towards higher cholesterol levels in the LP and OP groups compared with LC and OC groups (Table 5). The interaction between pain and weight groups for the cholesterol levels was not significant (cholesterol: *p=0.70*, HDL: *p=0.17*, LDL: *p=0.99*, and triglyceride: *p=0.96*).

## 4. Discussion

The novelty of this study resides in investigating the influence of musculoskeletal chronic pain as a factor by itself, or in combination with obesity, on sperm quality as a biomarker of fertility potential in men.

For the first time, we investigated sperm motility including detailed kinematic parameters, sperm morphology, DNA fragmentation, and reproductive hormone levels as biomarkers of male fertility in lean and overweight chronic pain patients compared with lean and overweight healthy controls.

Chronic pain and obesity have been advocated as independent risk factors for decreased serum testosterone levels and altered sperm fertility potential [32–35]. In chronic pain patients, the continuous nociceptive input can cause alterations in the central processing of nociception and maladaptive plasticity, resulting in increased pain sensitivity, which is manifested on lower PPT values [36]. Our study also demonstrated a tendency towards lower PPT values in the chronic pain groups (LP and OP) compared with their relative control groups (LC and OC), which is aligned with some of the previously conducted studies demonstrating decreased PPT values in chronic pain patients [36–40].

Chronic pain has also been considered as a drive for stress response, and this recurrent stressor effect on the HPA axis and GnRH axis has been demonstrated as a possible risk factor for testosterone decrease [18, 21, 41, 42]. Adequate testosterone levels have been shown to produce pain control in males since this hormone is intricately involved in endogenous opioid activity [40, 43]. Low levels of plasma testosterone result in elevated pain, which can be reflected on lower PPT values (higher muscle sensitivity), thereby suggesting that testosterone plays a role in pain modulation [18, 37, 43]. Despite previous reports supporting the role for testosterone in dampening pain and raising the pain threshold [44], no significant correlation was observed between chronic pain and the hormonal profiles in this study. However, a decreased testosterone level was found in the lean chronic pain patients (LP) compared with the related healthy matched controls (LC) that could explain—at least in part—some of our findings in the PPT assessments.

The demonstrated tendency for lower PPT values (higher sensitivity) in overweight chronic pain patients compared to overweight healthy matched controls supports the previous reports, suggesting that pain sensitivity may be mediated by sociocultural, psychological, and biological factors [45].

Androgens also play an essential regulatory role in spermatogenesis and the maturation and motility of the spermatozoa during their transportation through the epididymis and vas deferens [46]. It is known that sperm motility characteristics have an influence on the fertilization potential [31]. Furthermore, the sperm concentration has also been found to be clearly associated with fertility and conception rates [47]. Our results demonstrated that chronic pain patients (LP) have lower values of sperm motility parameters compared with the related healthy matched controls (LC). A significantly higher percentage of sperm with progressive motility in the LC compared to the OC group demonstrates the adverse effect of obesity on sperm motility similar to previous reports [48]. On the other hand, a significantly higher percentage of sperm with progressive motility in the LC compared to the LP group suggests a novel negative effect of chronic pain on sperm motility in the lean group. To date, there are a very limited number of studies where chronic pain, reproductive hormones, and sperm quality have been investigated together, and this limitation makes it difficult to compare or generalize the results of the present study. Results from a meta-analysis by Fu et al. on male fertility have indicated that chronic pelvic pain syndrome (CPPS) can significantly reduce sperm concentration and sperm kinematic parameters [41], which is in line with our findings in patients with musculoskeletal pain.

The OP group also demonstrates lower median values of progressively motile sperm compared with the OC group; however, the difference remained insignificant. Previous studies have similarly reported that obesity has a negative correlation with testosterone levels and consequently sperm quality parameters, leading to decreased fertility potential [7–9]. The adverse effects of obesity on reproductive hormones [49] and sperm motility could be considered as confounding factors while trying to assess the effect of chronic pain within the overweight groups affecting the significance of the reported results.

It may be speculated that even the insignificant change of testosterone levels observed in this study may have an effect on the maturation process of the sperm, thereby affecting the sperm motility and kinematic parameters. On the other hand, despite the demonstrated lower tendency in testosterone levels for chronic pain patients compared with the related healthy control group, it still remained within the (lower) reference range, and therefore, a significant difference and more conclusive results could be expected in a larger sample size. Prospective data are necessary to confirm these findings and explain the possible mechanism involved in the effects of chronic pain on male sperm quality, especially the kinematic parameters.

Collectively, our results indicate that chronic pain has no or minimal effect on sperm morphology and DNA fragmentation but significantly reduces the percentage of progressively motile sperm in lean men and exerts a negative effect on sperm concentration and kinematic parameters, which can be considered as valuable biomarkers of male fertility potential. However, the adverse overlapping effect of obesity on sperm quality makes it difficult to propose the exact effect of musculoskeletal chronic pain on sperm quality in overweight patients.

### 4.1. Strengths and Weaknesses

The newer generation of CASA systems used to assess detailed motility and kinematics of sperm samples in this study has the capability of providing more precise and objective results than previous generations [50, 51]. PPT measurements were performed by the same investigator to minimize variations. Pain intensity and quality, in the chronic pain subjects, were assessed by using PPT, VAS, and a modified version of the McGill Pain Questionnaire (MPQ) to give a multidimensional perspective and a better understanding of the pain characteristics in the recruited chronic pain patients. However, due to the limited sample size of this study, findings should be interpreted cautiously. Future studies with a larger sample size could provide more conclusive results in factors such as changes of hormonal levels. Confounding factors caused by the chronic pain, such as stress which is known to negatively affect sperm quality [52], should also be considered in future studies. Furthermore, measurement of some inflammatory and biochemical biomarkers could help clarify some of the potential underlying mechanisms.

## 5. Conclusion

While sample size of this study may not preclude a type 2 error, the results strongly suggest that musculoskeletal chronic pain has a significantly negative influence on sperm quality, seen as a significantly lower percentage of progressively motile sperm in lean men.

Despite significantly lower values of VCL, STR, and WOB in the overweight men with chronic pain compared to healthy controls, no significant difference in the percentage of sperm in different motility groups was observed which could be related to the overlapping negative influence of obesity on the sperm parameters. Further investigation to discriminate the effect of overweight and chronic pain in cases where both complications exist is suggested to confirm this hypothesis. Further studies are also warranted to assess the possible preventive or treatment strategies to break the link between obesity, chronic pain, and sperm quality.

## Figures and Tables

**Table 1:** The age and BMI (body mass index) of the participants in the study groups. Multivariate two-way analysis of variance (MANOVA) was used to compare differences in BMI and age.

	Age (years)		BMI	
	Mean ± SD	Median (min, max)	Mean ± SD	Median (min, max)
OP	30.8 ± 9.7	30 (18, 46)	29.7 ± 5.2	27.9 (25.2, 41.4)
OC	26.1 ± 3.8	25 (22, 33)	26.2 ± 1.5	25.6 (25.1, 29.8)
LP	27.6 ± 5.4	26 (19, 36)	23.7 ± 1.3	24.4 (20.9, 24.7)
LC	25.9 ± 6.9	23 (19, 42)	22.4 ± 0.5	22.4 (21.3, 22.9)

BMI: body mass index (kg/m^2^); OP: overweight with chronic pain; OC: overweight control; LP: lean with chronic pain; LC: lean control (N = 10 per group).

**Table 2:** Pressure pain threshold values in overweight pain patient (OP), overweight control patient (OC), lean pain patient (LP), and lean control patient (LC) in 16 different test points in the body. Provided values are mean ± standard deviation. Multivariate two-way analysis of variance (MANOVA) was used to compare differences in each parameter in pressure pain thresholds.

Group test point	LC		LP		OC		OP	
	Mean	Std. deviation	Mean	Std. deviation	Mean	Std. deviation	Mean	Std. deviation
p1: Vastus medialis muscle	386.29	134.97	231.84	103.78	366.24	155.36	211.37	154.97
p2: Rectus femoris	457.84	192.78	241.20	78.95	351.00	172.91	221.41	181.40
p3: Vastus lateralis muscle	369.40	221.56	227.64	82.77	325.52	210.58	190.75	177.75
p4: Adductor longus muscle	305.93	137.61	291.75	113.72	289.77	149.49	110.35	96.00
p5: Tibialis anterior muscle	355.74	197.83	244.14	107.21	277.60	161.21	178.05	149.39
p6: Peroneus longus muscle	404.23	198.05	247.66	102.08	304.76	174.70	227.83	144.37
p7: Patellar tendon	460.37	316.30	217.08	67.81	282.99	102.49	207.53	200.58
p8: pes anserinus bursae	316.84	174.43	225.36	82.27	276.56	155.86	200.82	112.23
p9: Popliteous muscle	332.07	197.19	272.26	90.40	295.79	161.76	244.15	168.62
p10: Iliacus muscle	399.08	91.24	244.86	133.52	318.13	135.21	228.00	149.85
p11: Quadrates lumborum muscle	283.61	124.37	233.43	66.02	254.01	186.66	227.16	102.97
p12: Supraspinous ligaments area between L5–S1 and S1–S2	304.00	154.53	289.28	109.48	296.07	170.83	217.15	119.43
p13: Extensor digitorum brevis	252.22	73.37	207.25	96.08	230.14	104.66	183.10	90.70
p14: Extensor digitorum brevis	317.94	128.46	239.44	126.77	242.30	74.64	224.30	146.16
p15: Extensor hallucis longus	297.27	173.54	198.99	49.99	256.87	188.01	245.38	62.91
p16: Flexor halluces longus	252.70	148.90	235.00	116.33	257.00	93.43	190.20	55.60
Control: Deltoids	358.38	128.38	244.91	126.80	260.58	100.78	232.87	110.40

None of the ANOVA results are significant.

**Table 3:** Sperm quality parameters in overweight with chronic pain (OP), overweight control (OC), lean with chronic pain (LP), and lean control (LC). Provided values are median (25–75 percentiles), and superscripted letters mark pairwise significant differences (*p*<0.05). Multivariate two-way analysis of variance (MANOVA) was used to compare differences in sperm quality parameters.

	OP	OC	LP	LC
Motility (WHO [31])				
Concentration (m/ml)	37.4 (31.9–92.3)	63.9 (45.3–78.5)	16.2 (8.0–50.3)	49.8 (23.9–100.9)
Immotile (%)	22.3 (13.0–44.5)	28.9 (19.6–42.7)	40.9 (39–48.7)	29 (12.1–57.0)
Nonprogressive motile (%)	50.5 (31.3–62.6)	26.35 (23.7–32.8)	28 (23.9–34.075)	22.85 (16.9–25.5)
Progressively motile (%)	24.2 (23.3–25.0)	39.5 (26.1–57.7)^b^	29.7 (22.8–33.4)^a^	42.6 (22.7–66.7)^a,b^
Hyperactivated sperm (%)	5.15 (2.0–10.4)	12.6 (5.9–17.6)	4.1 (1.8–4.6)	10.7 (4.7–24.1)
DNA fragmentation (%)	41.7 (30.3–65.3)	41.3 (28.1–66.7)	55.4 (39.2–72.0)	29 (16.5–76.1)
Normal morphology (%)	3.7 (3.1–6.5)	5 (2.7–13.8)	8 (7.0–9.9)	13.1 (7.4–20.6)

**Table 4:** Median (25–75 percentiles) kinematic parameters of sperm motility in overweight with chronic pain (OP), overweight control (OC), lean with chronic pain (LP), and lean control (LC) groups. Superscripted letters mark pairwise significant differences (*p*<0.05). Multivariate two-way analysis of variance (MANOVA) was used to compare differences in kinematic parameters.

Average kinematic parameters	OP	OC	LP	LC
VCL (*μ*m/s)	38.0 (27.2–39.4)^a^	42.8 (40.85–63.1)^a,b^	31.7 (28.4–36.3)	47.5 (31.1–54.1)^b^
VSL (*μ*m/s)	14.5 (11.2–21.5)	18.7 (14.55–29.8)	11.4 (10.3–14.1)	17.6 (12.2–21.2)
VAP (*μ*m/s)	20.9 (16.5–29.9)	25.5 (20.45–39.9)	17.0 (15.1–20.0)	24.2 (17.0–28.3)
LIN (%)	36.8 (31.6–43.9)	42.3 (35.6–46.0)	36.8 (34.5–40.1)	43.3 (36.8–46.8)
STR (%)	66.9 (63.1–72.8)^a,b^	73.3 (70.95–76.1)^a^	69.1 (63.5–70.8)^b^	75.0 (68.6–76.2)
WOB (%)	51.2 (45.0–54.8)^a,b^	57.3 (50.80–61.0)^a^	55.2 (53.2–56.3)^b^	56.8 (54.5–62.4)
ALH (*μ*m)	2.8 (2.6–3.8)	3.2 (2.90–3.3)	2.5 (2.30–3.0)	2.6 (2.3–2.9)
BCF (HZ)	5.1 (3.8–6.2)	6.5 (5.9–6.7)	4.8 (4.5–5.0)	5.6 (4.9–6.1)

**Table 5:** Lipid profile values in overweight pain patient (OP), overweight control patient (OC), lean pain patient (LP), and lean control patient (LC). Provided values are median (25–75 percentiles). Multivariate two-way analysis of variance (MANOVA) was used to compare differences in lipid profile.

	OP	OC	LP	LC
Cholesterol (mmol/L)	4.3 (3.7–5.0)	4.3 (3.4–5.0)	5.0 (4.6–5.5)	4.4 (3.9–5.9)
Triglyceride	0.7 (0.5–1.0)	1.0 (0.7–1.4)	1.2 (0.9–1.6)	0.7 (0.6–1.3)
LDL (mg/dl)	2.6 (2.0–3.2)	2.6 (1.8–3.2)	3.3 (2.6–3.6)	2.5 (2.1–4.2)
HDL (mg/dl)	1.5 (1.3–1.8)	1.3 (1.1–1.4)	1.3 (1.1–1.4)	1.2 (1.1–1.7)

None of the ANOVA results are significant.
